# The Effect of Hypertonic Saline Nebulization on Arterial Blood Gas Parameters Among Patients on a Mechanical Ventilator

**DOI:** 10.7759/cureus.66043

**Published:** 2024-08-02

**Authors:** Saraswathi M, Priyadharsini Masilamani, Kanniammal Chinnathambi

**Affiliations:** 1 Critical Care, Sri Ramaswamy Memorial (SRM) College of Nursing, Sri Ramaswamy Memorial (SRM) Institute of Science and Technology, Chengalpattu, IND; 2 Nursing, Sri Ramaswamy Memorial (SRM) College of Nursing, Sri Ramaswamy Memorial (SRM) Institute of Science and Technology, Chengalpattu, IND

**Keywords:** effect, arterial blood gas parameters., mechanical ventilator, 3% sodium chloride hypertonic saline (hts), nebulization

## Abstract

Introduction

Care of the airway is an essential part of the management of patients receiving mechanical ventilation. If the airway is not properly managed, an endotracheal airway can result in retained secretions, airway obstructions, and infections. These complications may prolong mechanical ventilation duration and length of hospital stay and may increase the cost of affordability. Hypertonic saline nebulized suctioning is a technique used to lessen the duration of mechanical air flow and enhance airway clearance, which helps patients on mechanical ventilation breathe easier.

Aim

The objective of the study is to assess the effectiveness of nebulization with hypertonic saline on arterial blood gas parameters among mechanically ventilated patients.

Methods

The quasi-experimental design adopted with thirty-five mechanically ventilated samples was chosen using a non-probability purposive sample technique. Following the pre-test in the endotracheal tube, nebulization was given with 2 ml of hypertonic saline over 15-20 mins, two times each day, to the mechanically ventilated patients. Post-test was carried out about 15-20 minutes after the procedure using arterial blood gas analysis results were obtained and interpreted.

Results

The study reveals that the p values corresponding to the arterial blood gas parameters PCo2, pO2, and HCo3 are less than 0.01 and are significant at a 1% level, and arterial blood gas (ABG) pH is less than 0.05 and is significant at a 5% level; hence there is a high significant difference between the pre-test and post-test mean scores of arterial blood gas parameters PCo2, pO2, HCo3, and ABG pH. Hence, the study concluded that nebulization with hypertonic saline for patients with mechanical ventilators is more effective in improving arterial blood gas parameters.

## Introduction

Care of the airway is an essential part of the management of patients receiving mechanical ventilation [1]. These complications may prolong mechanical ventilation duration and length of hospital stay and may increase the cost of affordability. Studies have been done over the years to improve airway management strategies [2]. Many of the studies emphasize the importance of airway management strategies and their impact on ventilator-associated events [3]. Mechanical ventilation is the commonly used intervention in intensive care units, such as airway management for patients [4].

A ventilator is a respiratory device that helps patients when they are unable to breathe by themself. An endotracheal tube is used as a port to connect the patient to the ventilator [5]. According to the NCBI Report of 2020, among 165 critically ill patients, 35.7% (59/165) of patients required mechanical ventilators [6]. Airway obstruction is common in mechanically ventilated patients due to various problems such as expiratory muscle weakness, mucociliary dysfunction, and unproductive cough. These causes may lead to endotracheal tube blockage that may be firm due to prescribed fluid therapy. The main goal of ICU is to minimize the duration of mechanical ventilation, as prolonged mechanical ventilation was proven to be associated with an increase in mortality and morbidity [7]. The goal of ICU is to reduce the duration of mechanical ventilation, as prolonged mechanical ventilation was proven to be associated with an increase in mortality and morbidity [8].

According to a study conducted by Haruna et al., 2022 among 400 samples, re-intubation is required for 51 (12.8%) [9]. If the airway is not properly managed, an endotracheal airway can result in retained secretions, airway obstructions, and infections. The most common reason for re-intubation was found to be difficulty in the excretion of secretions. Airway blockage will lead to poor oxygen perfusion in the body. Three essential steps of airway remodeling are humidification, nebulization, and suctioning. Airway clearance techniques will improve mucociliary function and improve the removal of secretions, inflammatory cells, and microorganisms [10]. Saline nebulization is a commonly used procedure to loosen the secretions and remove them easily from the endotracheal tube in intensive care units. Hypertonic saline nebulized suctioning has also proven to be effective in clearing airway secretions; however, it is not under regular use [10]. Generally, prescribed drugs for nebulization will be used to loosen the secretions [11]. Suctioning followed by nebulization with sodium-concentrated saline was demonstrated as an effective treatment to activate the mucociliary function, reduce the thickness of sputum, and reduce the swelling of the airway. It also prevents dryness in the airway. Care of the airway is an essential part of the management of patients receiving mechanical ventilation [12]. Airway blockage is directly proportional to gas exchange and acid-base imbalances. The worldwide incidence of people who require intensive care unit admission and mechanical ventilation is up to 20 million annually. Globally, according to Lance et al. (2015), the rate of seriously ill patients increased, and this study showed that around 4312 patients were admitted to three hundred ICUs within thirty-five nations, and among them, nearly 55% of patients were mechanically ventilated patients during intensive care unit admission.

Chakor et al. (2015) found that among the 1150 intensive care admissions, 3.91% of patients required prolonged ventilator support [13]. Also among 397 clients, 11.3% needed invasive ventilation. Weaning from mechanical ventilation is an essential element of caring for critically ill patients. So, the main focus of the health care provider is to wean the patient from a mechanical ventilator. Maintaining a patent airway has a major role in weaning the patient from the ventilator.

According to a study conducted by Sudarsanam et al., of the 200 mechanically ventilated patients, at discharge 143 patients (71.5%) had died due to various reasons such as respiratory failure, endotracheal tube obstruction, and so on [14]. In the present study, the investigator finds the effect of hypertonic saline nebulization on arterial blood gas parameters among mechanically ventilated patients.

## Materials and methods

The research adopted a quantitative evaluative approach with one group pre-test post-test design. In this study, the independent variable was nebulization with hypertonic saline, and the dependent variable was biophysiological measures and the level of airway clearance. The study was conducted in the ICU complex of Sri Ramaswamy Memorial (SRM) General Hospital, with a sample size of 35. The study included patients who were mechanically ventilated with age above 21 years. Patients who were admitted to the intensive care unit, patients' relatives who signed the informed consent, and patients who were discharged within three days were excluded from this study. Biophysiological measures of pre- and post-test values were collected using arterial blood gas (ABG) parameters (ABG-pH, ABG-pCo2, ABG-po2, ABG-HCo3). The validity obtained from experts in medicine and nursing from SRM General Hospital and Research Institute and SRM College of Nursing provided feedback on the tool's validity. The tool's suitability, relevance, and accuracy were confirmed through validation, and the Institutional Ethical Committee granted formal ethical approval. Data was collected from 35 mechanically ventilated patients chosen using a non-probability purposive sampling technique. Pre-test was done, and as an intervention, endotracheal tube nebulization was given for 15-20 minutes with 2 ml of hypertonic saline two times daily for three days. Followed by endotracheal suctioning performed approximately 5-10 seconds. Post-test was carried out about 15-20 minutes after the procedure by using arterial blood gas analysis.

## Results

This study concluded, with a total of 35 mechanical ventilator patients admitted to the ICU. Table 1 reveals that the p-values corresponding to the bio-physiological variables SPO2, PCo2, pO2, and HCo3 are less than 0.01 and are significant at 1% level hence there is a high significant difference between the pretest and post-test mean scores of bio-physiological variables SPO2, PCo2, pO2, and HCo3 also the p-value corresponding to the bio-physiological variable ABG pH is less than 0.05 and is significant at 5% level and hence there is a significant difference between the pretest and post-test mean scores of bio-physiological variable ABG pH.

**Table 1:** Clinical variables With regard to mechanical ventilation due to CNS disorder 14 (40%), whereas in normal saline group 12 (34.3%) also the reason for mechanical ventilation was CNS disorder. The maximum number of participants in both the hypertonic saline group and the normal saline group received second hourly suctioning, 21 (60%) and 22 (62.9%), respectively. Regarding the duration of mechanical ventilation in the hypertonic saline group majority of participants 19 (54.3%) were on mechanical ventilation for one week also in the normal saline group majority of patients were on mechanical ventilation for one week. Related to the patient's position during suctioning maximum patients both in hypertonic saline group 25 (71.4%) and in normal saline group 26 (74.3%) were in semi-Fowler's position. CNS - central nervous system

Clinical variables		Hypertonic saline	
		No	Percentage (%)
Reason for mechanical ventilation	CNS disorder	14	40
	Lung disorder	9	25.7
	Cardiac disorder	2	5.7
	Renal Disorder	3	8.6
	Metabolic disorder	7	20
Frequency of suctioning	Every 2 hours once	21	60
	Every 4 hours once	14	40
	Every 6 hours once	0	0
Duration of mechanical ventilation	One week	19	54.3
	One month	12	34.3
	More than one month	4	11.4
Patient position during suctioning	Supine position	10	28.6
	Semi-Fowler's position	25	71.4
Frequency of nebulization	Once a day	0	0
	Two times a day	35	100
	Three times a day	0	0
	Four times a day	0	0
Type of mucus secretion	Thick secretion	10	28.6
	Thin secretion	21	60
	Clear secretion	4	11.4

Table 2 explains the p-value corresponding to the bio-physiological variable SPO2 is less than 0.01 and is significant at 1% level hence there is a high significant difference between the post-test level mean scores of bio-physiological variable SPO2. Also, the p+values corresponding to the bio-physiological variables ABG pH, pCo2, pO2, and HCo3 are less than 0.05 and are significant at 5% level, hence, there is a significant difference between the post-test level mean scores of bio-physiological variables ABG pH, pCo2, pO2 and HCo3 of hypertonic saline nebulization.

**Table 2:** Comparison between the pre-test and post-test mean scores of arterial blood gas parameters ABG - arterial blood gas; ABG PH - arterial blood gas potential of hydrogen; PCo2 - partial pressure of carbon dioxide; PO2 - partial pressure of oxygen; HCo3 - bicarbonate ** Significant at 1% level; * Significant at 5% level

Bio Physiological variable	Test	N	Mean	SD	T value	Df	P value
ABG pH	Pretest	35	7.35	0.05	-2.148	34	0.039*
	Posttest	35	7.37	0.039			
pCo2	Pretest	35	46.17	4.662	2.824	34	0.008**
	Posttest	35	42.91	4.804			
pO2	Pretest	35	81.26	14.539	-4.499	34	>0.001**
	Posttest	35	93.49	14.251			
HCo3	Pretest	35	26.49	4.21	2.848	34	0.007**
	Posttest	35	24.17	3.959			

**Figure 1:**
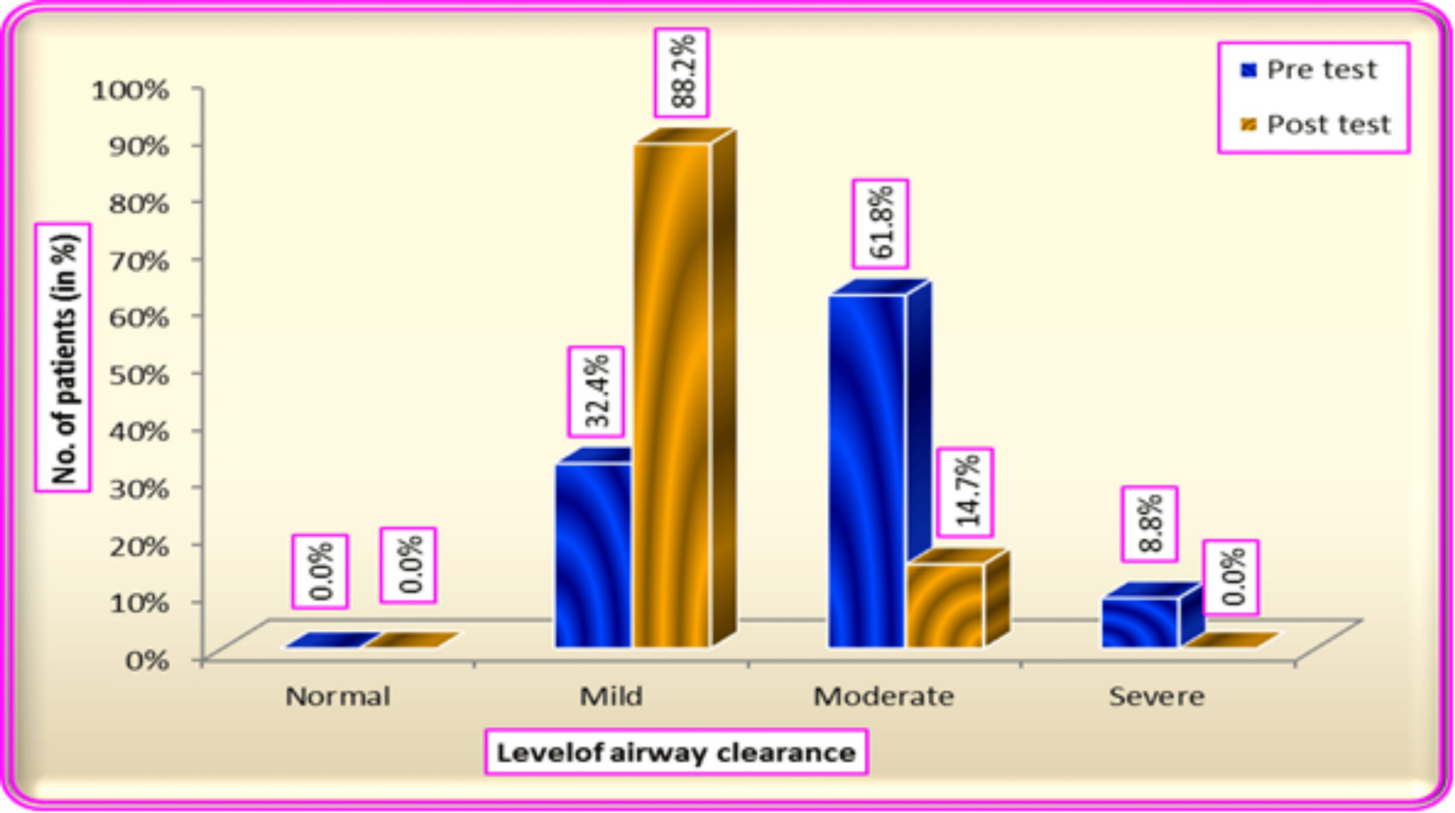
Results showing that hypertonic saline was effective in airway clearance at a p-value of 0.000 Percentage distribution of the level of airway clearance of mechanically ventilated patients among the hypertonic saline group

Table 3 shows in the pre-test 21 (61.8%) patients had moderate levels of airway clearance, 11 (32.4%) had mild levels of airway clearance and three (8.8%) had a severe level of airway clearance whereas the level of airway clearance improved in post-test as 30 (88.2%) had mild level of airway clearance and five (14.7%) had a moderate level of airway clearance after hypertonic saline nebulization. The study findings also show the frequency and percentage distribution of pre-test and post-test biophysiological variables, pre-test values related to heart rate 19 (54.3%) had 100-115, 13 (37.1%) had above 115 or below 70, three (8.6%) had 70-99, whereas in post-test, 22 (62.9%) had 70-99, 12 (34.3%) participants had 100-115 and one (2.9%) had above 115 or below 70. Related to SpO2 in the pre-test, 15 (42.9%) had 99%-85%, 13 (37.1%) had 100%-95% and seven (20%) had below 85%, whereas in the post-test, 25 (71.4%) had 100%-95%, 10 (28.6%) had 94%-85%. Regarding ETCO2 in the pre-test, 17 (48.6%) had 46-50, 14 (40%) had 35-45, and four (11.4%) had above 50, whereas in the post-test, 23 (65.7%) had 35-45, and 12 (34.3%) had 46-50. Related to ABG pH 16(45.7%) belonged to 7.30-7.34, 13 (37.1%) belonged to 7.35-7.46, and six (17.1%) were above 7.46 or below 7.30, whereas in the post-test, 24 (68.6%) had a pH of 7.35-7.46, 11 (31.4%) had 7.30-7.34. Related to pCo2 in the pre-test, 18 (51.4%) had 25-34, 12 (34.3%) had 35-46 and five (14.3%) had below 25 or above 46, whereas in the post-test, 24 (68.6%) had 35-46, nine (25.7%) had 34-25, and two (5.7%) had below 25 or above 46. Po2 in the pre-test, 15 (42.9%) had 70-89, 10 (28.6%) each in above 90 and below 70, whereas in the post-test, 21 (60%) had above 90, 12 (34.3%) had 70-89, and two (5.7%) had below 70. Related to HCO3 in the pre-test, 16 (45.7%) had 27-35, 13(37.1%) had 17-26 and 6(17.1%) had above 35 or below 17, whereas in the post-test, 23 (65.7%) had 17-26 and 12 (34.3%) had 27-35. Table 4 shows the distribution of the level of airway clearance of mechanically ventilated patients, and Figure 1 shows results that hypertonic saline was effective in airway clearance at a p-value of 0.000.

**Table 3:** Post-test mean scores of bio-physiological variables among the hypertonic saline group SPO2 - saturation of peripheral oxygen; ETCO2 - end-tidal carbon dioxide; ABG pH - arterial blood gas potential of hydrogen; pCo2 - partial pressure of carbon dioxide; pO2 - partial pressure of oxygen; HCo3 - bicarbonate ** Significant at 1% level; * Significant at 5% level

Biophysiological variable	N	Mean	SD	T value	Df	p-value
Heart rate	35	91.26	14.082	1.816	68	0.074
SPO2	35	96.03	3.944	-4.107	68	>0.001**
ETCO2	35	35.94	6.301	-0.173	68	0.863
ABG pH	35	7.37	0.039	-2.485	68	0.015*
pCo2	35	42.91	4.804	1.999	68	0.049*
pO2	35	93.49	14.251	-2.618	68	0.011*
HCo3	35	24.17	3.959	2.232	68	0.029*

**Table 4:** Distribution of the level of airway clearance of mechanically ventilated patients The table shows that in the pre-test, 21 (61.8%) patients had a moderate level of airway clearance, 11 (32.4%) had a mild level of airway clearance and three (8.8%) had a severe level of airway clearance, whereas the level of airway clearance improved in post-test as 30 (88.2%) had mild level of airway clearance and five (14.7%) had a moderate level of airway clearance after hypertonic saline nebulization.

Level of airway clearance	Hypertonic saline nebulization			
	Pre-test	%	Post-test	%
Normal	0	0	0	0
Mild	11	32.4	30	88.2
Moderate	21	61.8	5	14.7
Severe	3	8.8	0	0

## Discussion

In the endotracheal tube, nebulization was given with 2 ml of hypertonic saline over 15-20 mins, two times each day for three days. Followed by endotracheal suctioning performed approximately 5-10 seconds for the patients. The patients who were discharged within three days were excluded from this study. The post-test was carried out about 15-20 minutes after the procedure. Biophysiological measurements were obtained from a continuous cardiac monitor, and arterial blood gas analysis reports were obtained. The level of airway clearance was recorded before and after the administration of hypertonic saline nebulized suctioning. Hypertonic saline nebulization is cheap, safe, and effective in maintaining the airway patency of patients who are connected to an artificial airway. The major findings of the study were there is a highly significant difference between the pre-test and post-test mean scores of airway clearance at the p-value >0.001, and is significant at a 1% level. The mean value at the post-test level is less than the mean value at the pre-test level, and the study concludes that hypertonic saline is effective in airway clearance. The p-values corresponding to the biophysiological variables SPO2, PCo2, pO2, and HCo3 are less than 0.01 and are significant at a 1% level; hence, there is a high significant difference between the pre-test and post-test mean scores of bio-physiological variables SPO2, PCo2, pO2, and HCo3 of hypertonic saline nebulization.

## Conclusions

The study concludes that the effectiveness of nebulization with hypertonic saline on biophysiological measures and the level of airway clearance among mechanically ventilated patients. The study also concludes that the nebulization with hypertonic saline for patients with mechanical ventilators is more effective in maintaining arterial blood gas parameter values like SPO2 and pO2, reducing pCO2, and maintaining pH at normal range, and also more effective on airway clearance. The nurse has the primary role in maintaining a patent airway for mechanically ventilated patients. So, it is important to improve their knowledge and practice related to airway clearance. This can be enabled by applying the research into the practice.

## Appendices

Tool for data collection:

Socio-demographic variables:

1.   Age in years

a.       21-30

b.      31-40

c.       41-50

d.      51-60

e.      60 and above

2.   Gender

a.      Male

b.      Female

c.      Others

3.   Occupational status

a.      Business

b.      Daily wages

c.      Unemployed

d.      Private

e.      Government

4.   Income

a.      Rs<1000

b.      Rs 1000- 3999

c.      Rs 4000- 6999

d.      Rs 7000- 9999

e.      Rs>10000

5.   Type of family

a.       Nuclear family

b.      Joint family

c.       Extended family

6.   Dietary pattern

a.      Vegetarian

b.      Non-vegetarian

7.   Personal habits

a.      Alcoholic

b.      Smoking

c.      Tobacco chewing

d.      Any two of the above

e.      None

Clinical Variables:

1.   Reason for mechanical ventilation

a.      CNS Disorder

b.      Lung disorder

c.      Cardiac disorder

d.     Renal disorder

e.      Metabolic disorder

2.   Frequency of suctioning

a.      Every 2nd hourly once

b.      Every 4th hourly once

c.      Every 6th hourly once

3.   Duration of mechanical ventilation

a.      One week

b.      One month

c.      More than one month

4.   Patient Position during suctioning

a.      supine position

b.      semi-Fowler's position

5.   Frequency of Nebulization

a.      once a day

b.      two times a day

c.      three times a day

d.     four times a day

6.   Type of mucous secretion

a.      thick secretion

b.      Thin secretion

c.      clear secretion

7. Biophysiological parameters:

a.      Heart rate

b.      Spo2

c.       ETCO2

d.      ABG-Ph

e.       ABG-PCo2

f.       ABG- Po2

g.      ABG- HCo3

Level of airway clearance tool

1. Heart rate 70-99 scored as 0; 100-129 scored as 1; above 115 scored or below 70 scored as 2

2. Spo2 100-95 - 0; 94-85 - 1; below 85- 2

3. Etco2 30-45 - 0; 46-50 - 1; above 50 or below 30 - 2

4. ABG ph 7.35-7.46 - 0; 7.30-7.34 - 1; Above 7.46 or Below 7.30 - 2

5. pCo2 35-46 - 0; 34-25 - 1; Above 46 or below 25 -2

6. pO2 Above 90 - 0; 70-89 - 1; Below 70 - 2

7. HC03 17-26 - 0; 27-35 -1; Above 35 or below 17 - 2
